# The effects of mild traumatic brain injury induced by a low‐intensity blast exposure on phosphoproteome networks and cognitive function influenced by mutant tau overexpression

**DOI:** 10.1002/alz.084781

**Published:** 2025-01-03

**Authors:** Zezong Gu, Marcus Jackson, Shanyan Chen, Thi Thao Nguyen, Heather R. Siedhoff, Ashley Balderrama, Amitai Zuckerman, Runting Li, C. Michael Greenlief, Gregory M Cole, Sally A. Frautschy, Jiankun Cui

**Affiliations:** ^1^ University of Missouri, Columbia, MO USA; ^2^ Harry S. Truman Memorial Veterans’ Hospital Research Service, Columbia, MO USA; ^3^ Veterans Greater Los Angeles Healthcare System, Los Angeles, CA USA; ^4^ University of California Los Angeles, Los Angeles, CA USA

## Abstract

**Background:**

This study was to elucidate the impact of blast‐induced neurotrauma (BINT) on phosphoproteome networks and cognition in a genetically heterogeneous population of mice (rTg4510) with the human tau P301L mutation linked to Alzheimer’s disease‐related dementia (ADRD) including frontotemporal dementia.

**Method:**

Mild traumatic brain injury was induced in rTg4510 mice exposed to a single low‐density blast (LIB) at an upright position. After assessment of cognitive function by the automated‐Home Cage Monitoring (aHCM) system, frontal cortex tissue was collected at 40 days post‐injury. The label‐free tandem mass spectrometry using the data‐independent acquisition and parallel accumulation‐serial fragmentation techniques for quantitative proteomics and weighted peptide co‐expression‐network analysis (WpCNA) approaches were used to evaluate phosphoproteomes in association with synaptic function and learning ability.

**Result:**

In this study, blast‐exposed rTg4510 mice demonstrated a lower learning index, using the CognitionWall test by aHCM, compared to all other groups including non‐carrier unexposed sham controls. Among total 17,637 phosphopeptides identified, 706 and 550 were significantly changed in rTg4510 and non‐carrier LIB‐exposed mice relative to unexposed sham controls, respectively. Using WpCNA, we found phosphopeptide networks tied to associative learning and mossy‐fiber pathways, which predicted learning outcomes. Phosphopeptide expression in these networks were inversely related to learning and linked to synaptic dysfunction, cognitive decline, and dementia. LIB selectively increased pSer262 Tau in rTg4510, a site implicated in initiating tauopathy.

**Conclusion:**

This study unveils the relationship between ADRD genetic susceptibility, BINT, and cognitive decline, thus offering potential pathways as therapeutic targets for precision medicine to alleviate the disease burden among those affected by BINT.

